# Examining the Effect of Knowledge Seeking on Knowledge Contribution in Q&A Communities

**DOI:** 10.3390/bs14090853

**Published:** 2024-09-23

**Authors:** Junping Qiu, Qinze Mi, Zhongyang Xu, Shihao Ma, Yutian Fu, Tingyong Zhang

**Affiliations:** 1 School of Management, Hangzhou Dianzi University, Hangzhou 310018, China; jpqiu@hdu.edu.cn (J.Q.); hdumqz@hdu.edu.cn (Q.M.); hdufuyutian@hdu.edu.cn (Y.F.); hdglxyzty@hdu.edu.cn (T.Z.); 2 Chinese Academy of Science and Education Evaluation, Hangzhou Dianzi University, Hangzhou 310018, China; 3 School of Information Management, Nanjing University, Nanjing 210023, China; 4 Key Laboratory of Adolescent Cyberpsychology and Behavior, Ministry of Education, Wuhan 430079, China; mashihao@mails.ccnu.edu.cn; 5 School of Psychology, Central China Normal University, Wuhan 430079, China

**Keywords:** Q&A communities, knowledge seeking, knowledge contribution, motivational theory

## Abstract

Based on motivational theory, this study investigated the effect of users’ knowledge seeking on users’ knowledge contribution in question-and-answer (Q&A) communities. We collected 4643 samples from the largest social Q&A platform in China (Zhihu) and applied a mediation effect test to the data. The results showed that knowledge seeking affects intrinsic motivations (altruism and self-efficacy) and extrinsic motivations (social support, group identity, and reputation), further affecting knowledge contribution. Our findings indicated that Q&A communities should be concerned with users’ intrinsic and extrinsic motivations to ensure balanced knowledge exchange on social Q&A platforms, ultimately fostering long-term stability and growth. Existing research has mainly focused on a single behavioral state, such as knowledge seeking or knowledge contribution, and has paid little attention to the connection between these two types of user information behaviors. This study aimed to fill this gap by revealing the mechanisms through which users’ knowledge seeking affects their knowledge contribution.

## 1. Introduction

When people encounter difficulties in their lives, they may seek help in question-and-answer (Q&A) communities. Q&A communities provide an important platform for knowledge seeking and contribution among users [1]. These communities have improved their functions and present users with a compelling experience. For example, social functions have been added to provide personalized solutions to user problems [2]. A representative example is Zhihu, a reputable Q&A community in China. During its initial stage (2011–2013), Zhihu adopted an invitation system aimed at IT professionals, who contributed high-quality answers. In the development stage (2014–2016), Zhihu opened its registration to the public. Many users flooded the platform, and it rapidly expanded its user base. In the transformation stage (2017–2019), Zhihu strengthened the reviewing and screening of content and tried to improve knowledge quality. It also launched many columns, such as Zhihu Live and Zhihu Matrix. Recently (2020–), Zhihu has continued to develop new Q&A and social functions, such as Zhihu Video and Zhihu Bookstore. Zhihu has evolved into the largest comprehensive knowledge Q&A platform in China, covering various fields of society [3].

Q&A communities face a common problem: most users are knowledge seekers, and there are few knowledge contributors. Zhao et al. [4] noted that about 80% of users are knowledge seekers, while knowledge contributors account for only 20%. Moreover, many knowledge contributors have no subsequent contribution behavior after their initial contributions [5]. More knowledge contributors can enhance the breadth and depth of platform content and facilitate knowledge renewal, especially when there are more professional contributors [3]. Owing to their in-depth research in specific fields, they can significantly improve the quality of answers. High-quality content and a vibrant community atmosphere can attract more users, increasing the traffic on social Q&A platforms [3]. Therefore, enhancing users’ intentions to contribute knowledge has become a critical concern for Q&A communities [6]. Previous studies have focused on user knowledge contributions. Zhou et al. [7] adopted social cognitive theory to examine knowledge contribution in open-source communities. Deng et al. [8] used the configuration method to explore users’ knowledge contributions in online health communities. In comparison, little attention has been paid to the relationship between knowledge seeking and knowledge contribution. “Knowledge seekers and knowledge contributors are divided into two camps” [4].

However, Q&A communities have large user bases, with some members serving as knowledge seekers while also providing or sharing their skills and knowledge with other users on the platform. Frequent knowledge seeking may improve users’ self-efficacy, enrich their knowledge reserves, and make them feel capable and obligated to contribute knowledge to the community. In other words, knowledge seeking may impact knowledge contribution, which will contribute to developing Q&A communities. Therefore, the research question is as follows: What is the mechanism of user knowledge seeking on user knowledge contribution in Q&A communities? This study draws on motivational theory to investigate the two behaviors of users in Q&A communities. The research findings will enhance our understanding of user knowledge behavior and promote the sustainable and rapid development of Q&A communities.

The rest of this paper is organized as follows. In Section 2, we review the relevant literature. In Section 3, we present the research model and hypotheses, and describe the complete data collection and analysis process. In Section 4, we further discuss the results and present implications and limitations. In Section 5, we summarize our conclusions.

## 2. Literature Review

### 2.1. Q&A Community User Behavior

Q&A communities are Web 2.0-based platforms for users to exchange knowledge through Q&A [9]. As emerging applications, Q&A communities have attracted much interest from scholars investigating various dimensions of user behavior and interaction within these platforms. Scholarly research helps us understand user information needs and preferences, supports platform optimization, and promotes knowledge sharing and community development. We found that studies mainly focus on users’ knowledge payment (users pay for access to knowledge) [10,11], knowledge adoption (users accept shared knowledge) [12], answerer selection (users choose responders to answer) [13], and other behaviors in Q&A communities [14].

Zhao et al. [10] analyzed users’ knowledge of payments based on social capital theory. Their results showed that the number of articles written by knowledge contributors, the number of followers, the number of questions answered, and the number of published articles positively impact knowledge payment. Zhao et al. [11] investigated how knowledge seekers switch from free to paid platforms. They found that economic costs and cognitive locking negatively affect switching behavior, whereas perceived relative advantage, financial benefits, and subjective norms have positive effects. Li et al. [12] studied the influence of horizontal and vertical answer deviations on users’ knowledge adoption, further revealing the relevant interaction mechanism between static and situational factors in knowledge adoption. Zhang et al. [13] investigated the answerer selection behavior of knowledge seekers in Q&A communities. They noted that the professionalism and popularity of respondents, Q&A information, and service quality significantly affect the selection behavior of knowledge seekers. Li et al. [14] identified the linguistic characteristics that affect response quantity in Q&A sites, including sadness, positive emotion, and second-person pronouns.

There was a scarcity of research examining the impact of users’ knowledge seeking on their knowledge contribution. This study attempted to fill this gap by uncovering how knowledge seeking influences knowledge contribution.

### 2.2. Motivational Theory

Motivation refers to an intrinsic or extrinsic tendency toward activity [15]. Human behavior is driven by motivations, including intrinsic motivations and extrinsic motivations [16]. 

Intrinsic motivations are generated by intrinsic satisfaction, ideas, and desires to do something [17]. When people can decide their behaviors independently, intrinsic motivations are a powerful source driving their behaviors [18]. Altruism (AL) is an individual’s psychological motivation to help others [19]. People derive pleasure and satisfaction because their behavior helps others. Chen et al. [20] noted that altruism reflects a user’s social mission and responsibility. Yin et al. [21] examined user participation in Weibo topic discussions and found that altruism promotes users’ intentions to participate in Q&A communities. Geng et al. [22] surveyed Q&A communities in China, the US, and India and reported that altruism facilitates user knowledge sharing behavior in these three countries. Self-efficacy (SE) reflects an individual’s assessment of their ability to conduct a certain behavior [23]. In Q&A communities, self-efficacy reflects a user’s confidence in their ability to provide useful knowledge to others [24]. Burmeister et al. [25] noted that self-efficacy can enhance users’ identity centrality, positively influencing knowledge sharing behaviors. Keshavarz et al. [26] noted the effect of self-efficacy on online information evaluation. 

Extrinsic motivations mean that completing something will produce meaningful results, such as receiving more likes, gaining more followers, receiving honorary titles, or making work more interesting [27]. Social support (SS) refers to the respect and concerns expressed by others [28]. Social support includes informational support and emotional support [29]. Informational support emphasizes answering users’ questions through knowledge or information, while emotional support emphasizes indirectly helping users solve problems through listening, caring, and other methods [30]. Zhou et al. [31] investigated users’ knowledge sharing in online health communities and noted that social support affects knowledge sharing through subjective norms. Wang [32] examined users’ obtaining health information and found that the less support they receive in real society, the more likely they are to seek social support from virtual communities. Group identity (GI) refers to a specific group of users’ overall evaluation of the products they use [33]. A few studies have applied group identity to the “object”. That is, when a user rates a certain object (such as a movie), other users will form their initial impression based on this rating. Lee et al. [34] argued that group identity can also be applied to the “person”, which means that a specific group of users has a sense of identity with the group values and an emotional attachment to the group. Cui et al. [35] noted the impact of group identity on users’ continuous contribution behaviors. Reputation (RE) reflects the honor and reward an individual receives from social activities. It is different from tangible incentives such as money [36]. In Q&A communities, users gain recognition and respect from other users by sharing information and knowledge, and possessing a certain level of virtual status on the platform can bring them many benefits [37]. Zhang et al. [38] emphasize that users may answer questions in online environments to demonstrate their expertise in a particular field, thereby gaining reputation. Nguyen et al. [39] noted that reputation positively correlates with online knowledge sharing behavior. 

Prior studies have utilized motivational theory as a framework to delve into user behavior within online communities, including altruism, self-efficacy, social support, group identity, and reputation. However, no literature currently applied motivational theory to the relationship between the knowledge seeking and knowledge contribution. This paper will make up for this deficiency.

### 2.3. Knowledge Seeking and Knowledge Contribution

Knowledge seeking (KS) is when users use search functions to obtain knowledge that meets their knowledge needs [40]. Knowledge seeking requires users to invest the necessary time and effort, and it is often deemed an active participation behavior [41]. Knowledge seeking not only helps users become continuous learners but also stimulates the exploration desires of other users. This may promote the use of Q&A communities. Fu et al. [42] noted that information adoption, usefulness, and satisfaction have an impact on continuous seeking, but Jin et al. [43] found that self-presentation negatively impacts knowledge seeking behavior.

Knowledge contribution (KC) means that community users transmit their accumulated knowledge and experience to other users [44]. Transmission methods include writing articles, replying to comments, or editing existing public information [45]. Knowledge contribution expands the total knowledge in Q&A communities and provides a continuous power source for developing these websites. It can also update outdated knowledge. Zhou et al. [46] noted that both social participation and social recognition have a positive impact on users’ knowledge contributions to online Q&A communities. Luo et al. [47] studied the continuous knowledge contribution of users in social Q&A communities from the perspective of user self-presentation and motivational affordances. Zhang et al. [48] examined knowledge contribution behavior in social knowledge communities from the perspective of social distance. 

Previous studies have indicated a direct relationship between knowledge seeking and knowledge contribution. Park et al. [49] suggest that knowledge seeking can influence knowledge sharing through mediating variables. Furthermore, Wang et al. [1] have shown that knowledge seeking can affect knowledge contribution through three mediating variables: Structural Capital, Relational Capital, and Cognitive Capital. Additionally, Luo et al. [47] emphasize that knowledge seeking, along with other variables such as social learning and peer recognition, can directly influence knowledge contribution. This paper will not only examine the direct relationship between the two but will also introduce the framework of motivational theory to assess the indirect relationship between them. We aim to address the gap in the existing literature, which primarily focuses on a single behavior.

## 3. Data Collection and Analysis

### 3.1. Research Hypotheses and Models

In the previous text, we highlighted that both intrinsic and extrinsic motivations are important drivers for changes in user behavior. From an intrinsic motivation perspective, when users receive answers from others in a Q&A community, they may develop a sense of obligation to help in return, driven by a psychological motivation to repay. This enhances altruism. Through mutual interactions, users create value and assist others in making decisions by contributing knowledge. They find helping others both meaningful and enjoyable, which further encourages their knowledge contribution behavior. At the same time, frequent knowledge seeking activities in Q&A communities can improve users’ ability to find information effectively. As users become more confident in their knowledge seeking skills, they may feel that they possess sufficient expertise to help others and solve problems for other users. This reinforces their willingness to contribute knowledge. 

From an extrinsic motivation perspective, when users post questions in a Q&A community, they often receive informational or emotional support from other members. This fosters a sense of social support, which encourages users to become more involved in the community and build emotional connections such as trust and attachment. These emotional bonds promote further knowledge contribution. As users seek answers, they also engage in social interactions, forming relationships and networks with other members. Users who interact with them may follow the topics they discuss and share common interests. This can cultivate a sense of belonging and develop group identity, which encourages knowledge contribution behavior. Additionally, users may raise meaningful, in-depth, and insightful questions during their knowledge seeking, attracting attention and sparking discussions. This can enhance their influence and reputation. Once they establish a certain level of reputation, users may actively contribute more knowledge to further boost their reputation and expand their influence. Therefore, we proposed the following hypotheses.

**H1.**  *Knowledge seeking positively affects altruism.*

**H2.**  *Altruism positively affects knowledge contribution.*

**H3.**  *Knowledge seeking positively affects self-efficacy.*

**H4.**  *Self-efficacy positively affects knowledge contribution.*

**H5.**  *Knowledge seeking positively affects social support.*

**H6.**  *Social support positively affects knowledge contribution.*

**H7.**  *Knowledge seeking positively affects group identity.*

**H8.**  *Group identity positively affects knowledge contribution.*

**H9.**  *Knowledge seeking positively affects reputation.*

**H10.**  *Reputation positively affects knowledge contribution.*

Meanwhile, the process of seeking knowledge simultaneously increases the users’ knowledge reserves, enabling them to contribute knowledge through their actions as a way to give back to the community. Therefore, we hypothesize that:

**H11.**  *Knowledge seeking can directly or indirectly positively affect knowledge contribution.*

**Figure 1:**
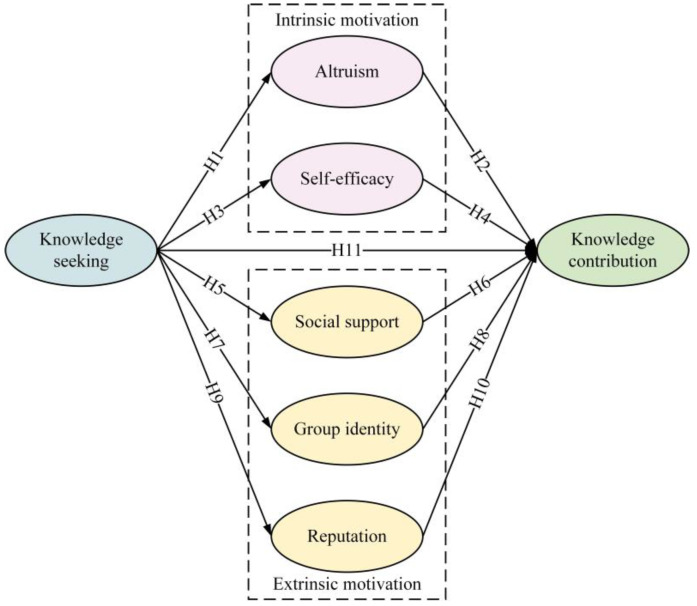
Research model.

Figure 1 presents the model. Intrinsic motivations include altruism and self-efficacy, whereas extrinsic motivations include social support, group identity, and reputation.

### 3.2. Data Collection

**Figure 2:**
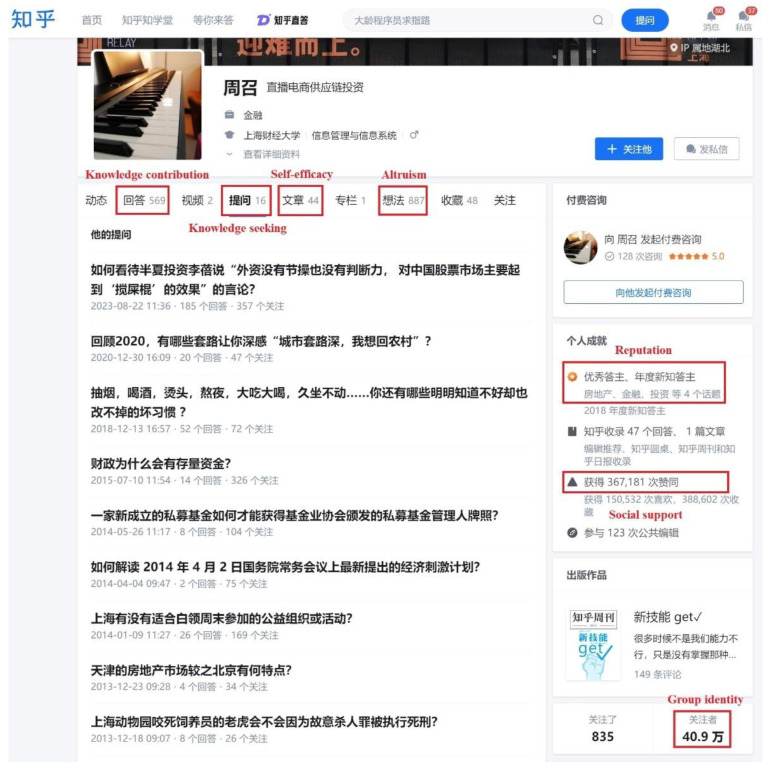
A screenshot of a Zhihu user’s homepage.

This research uses Zhihu, a well-known Q&A community in China, as the research object. Indicators measuring variables were adapted from the literature to improve the content validity. On the one hand, knowledge seeking is measured by the number of questions asked by a user, whereas knowledge contribution is measured by the number of answers posted by a user [1]. On the other hand, on the Zhihu platform, users can post “ideas” and “articles”. “Ideas” are usually short topics, and users post them to attract discussion and attention by sharing their opinions to help others. People with more significant altruism will post more valuable ideas [50]. An “article” is usually a long piece of content posted on the platform, mainly containing detailed analysis or research. By posting “articles”, users can better export and disseminate knowledge, gain recognition, and thus improve their confidence and self-efficacy [24]. Therefore, in this study, altruism is reflected by the number of ideas updated by a user [35], reflecting the user actively outputting viewpoints and information beneficial to others on social Q&A platforms. Self-efficacy is reflected by the number of articles updated by a user, which are closely related to the user’s confidence in their knowledge and experiences [51]. Social support is measured by the number of likes received by a user, reflecting mutual support and encouragement between community members. This has been found to encourage members to engage in collective knowledge sharing and discussions [52]. Group identity is reflected by the number of followers a user has, representing attention and recognition from the social network established on a platform [53]. Reputation is measured by the user’s number of good answer titles [54], which not only show their expertise and contributions on the platform but also reflect a high level of respect from other members. Figure 2 shows the location of the above measurements on Zhihu.

After determining the measurement indicators, we randomly selected 13,814 accounts on the Zhihu platform. Some were deactivated; some did not involve Q&A; and some belonged to official accounts (e.g., Zhihu Daily News, Zhihu Video, etc.), which are not the subject of this study. Based on these findings and the research results of Li et al. [12] and Jin et al. [43], we applied the following criteria to filter the sample: First, we excluded official and deactivated accounts. Second, we ensured that the number of questions and answers was greater than zero. Third, we confirmed that at least one of the remaining five indicators in our sample must be greater than zero. After screening, we obtained a sample of 4643 active users. Table 1 lists the descriptive statistics of the variables.

Table 1 showed that, except for reputation, the standard deviation of the factors was significantly large. Thus, natural logarithm treatment was used to eliminate the impact of different dimensions and magnitudes on model fitting (rounding to two decimal places) [7,12].

### 3.3. Data Analysis

**Figure 3:**
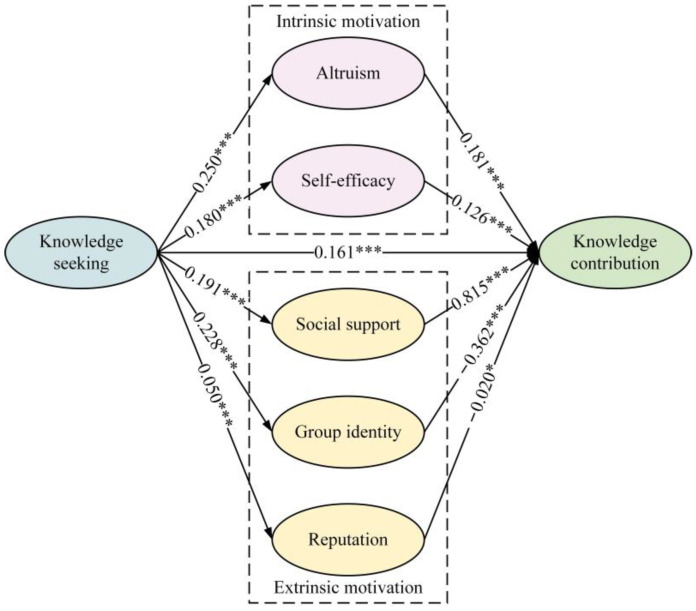
Parallel mediation between knowledge seeking and knowledge contribution. Notes: * *p* < 0.05, *** *p* < 0.001; *β* is standardized.

To ensure the reliability of the results, standardization, and completeness of the testing process, we strictly adhered to the mediation effect analysis procedures proposed by Xu et al. [55] and Li et al. [56] for testing the parallel mediation model depicted in Figure 1. In this study, we employed PROCESS, an SPSS macro developed by Hayes [57], specifically Model 4, to test the mediating effects of AL, SE, GI, RE, and SS between KS and KC. The confidence intervals were 95%, and bias-corrected bootstrapping was performed with 5000 samples. The outcomes are presented in Table 2, and illustrated in Figure 3.

The results in Table 2 indicated that KS significantly and positively predicts AL (β = 0.250, *p* < 0.001), SE (β = 0.180, *p* < 0.001), SS (β = 0.191, *p* < 0.001), GI (β = 0.228, *p* < 0.001), and RE (β = 0.050, *p* < 0.001). KS has a direct impact on KC (β = 0.161, *p* < 0.001). In addition, AL (β = 0.181, *p* < 0.001), SE (β = 0.126, *p* < 0.001), and SS (β = 0.815, *p* < 0.001) all significantly and positively predict KC, whereas GI (β = −0.362, *p* < 0.001) and RE (β = −0.020 *p* < 0.05) have significant negative predictive effects on KC. It was worth noting that the results of the data analysis in Table 2 indicated that the *R*^2^ and β values were not large. The reason for this phenomenon was that there were various influencing factors in the process of knowledge seeking on knowledge contribution. “In social sciences, *R*^2^ is usually low, especially in cross-sectional data analysis [58]”. This study aimed to explore only the influential role and mediating effect of motivational factors and not all the influential factors in this process, so the *R*^2^ and *β* values were within the acceptable range in this study.

Figure 3 showed the testing model for the parallel mediation effect between knowledge seeking and knowledge contribution. Table 3 shows that except for KS → RE → KC, the 95% Bootstrap confidence intervals for the remaining four mediating paths do not include 0. This indicates that the mediating effect of reputation between knowledge seeking and knowledge contribution is insignificant (indirect effect = −0.001, Boot SE = 0.001, 95% CI [−0.003, 0.000]), while the other four pathways exhibit parallel mediating effects. Among these, the mediating effect size for AL is 0.045, for SE is 0.023, and for GI is −0.083. Not only is the effect size of GI larger than those for AL and SE, but its effect also runs counter to the direction of the direct effect, suggesting a ‘masking effect’ or ‘counteractive effect’. It is worth emphasizing that we found the mediating effect of social support to be 0.156 (Boot SE = 0.014, 95% CI [0.159, 0.215]), which is the highest value observed in the analysis. This indicates that social support plays a significant role in the relationship between knowledge seeking and knowledge contribution. Finally, by summing all the mediation effect values, we obtained a total indirect effect value of 0.140 (Boot SE = 0.013, 95% CI [0.142, 0.194]). Additionally, this study identifies the total effect of KS on KC as 0.301. (Boot SE = 0.017, 95% CI [0.328, 0.393]).

To demonstrate the optimality of our model and address the issue of data directionality, we examined the impact of knowledge contribution on knowledge seeking through mediating variables. The results are shown in Table 4.

Under the comparison of the two models, the total indirect effect in the reverse test model is negative (total indirect effect = −0.025, Boot SE = 0.015, 95% CI [−0.056, 0.006]), and the 95% CI includes 0. This indicates that the results of the reverse model are not ideal. In other words, the model which knowledge seeking influences knowledge contribution through the mediating variable is the optimal model.

## 4. Discussion

### 4.1. Summary of Results

The results of the mediation effect test showed that all 9 hypotheses, H1, H2, H3, H4, H5, H6, H7, H9, and H11, were supported, except for hypotheses H8 and H10, which were not supported.

The key finding of this study is that social support plays a significant mediating role in the relationship between knowledge seeking and knowledge contribution, accounting for the majority of the effect (51.69%). When users ask questions on a social Q&A platform, the platform’s algorithm typically displays the questions to many unfamiliar users. When these users interact with the questioner through likes, the questioner realizes that their question is not only useful to themselves but also beneficial to others. This form of social support can effectively reduce the sense of unfamiliarity for the knowledge seeker, making them aware that they are not just information seekers but also part of the community. As the number of likes increases, the social support from the community helps users gradually develop a sense of belonging. They no longer see themselves as outsiders but rather as active members of the community. This sense of belonging not only brings positive emotional changes on a psychological level but also translates into actual behavior. After receiving positive feedback, users are more motivated to engage with the platform, contributing their own knowledge by organizing what they already know and what they have learned from the Q&A community.

In addition, the results showed that users’ knowledge seeking behavior enhances altruism and self-efficacy; at the same time, both intrinsic motivations promote this behavior. Combining existing studies and realistic scenarios, we can see that the reason for this phenomenon is that, on the one hand, when a user engages in knowledge seeking behavior, they are receiving help from others and may feel grateful to the Zhihu platform and society. They may develop altruism to give back to society and help others, thus exhibiting knowledge contribution behaviors [59]. On the other hand, when users frequently engage in knowledge seeking behaviors in the community, they expand their knowledge reserve to obtain the answers they want by continuously learning and absorbing others’ knowledge. With the increased knowledge reserve, they develop a certain sense of self-efficacy and believe they can help others solve problems—i.e., “the greater the ability, the greater the responsibility”—and then switch from being knowledge seekers to contributors [60]. These discussions can be further supported by qualitative methods in future research.

On the other hand, we found that knowledge seeking positively affects group identity, but group identity negatively affects users’ knowledge contributions. This result was contrary to previous studies, which reported that group identity has a positive impact on knowledge sharing [61]. In a realistic scenario, this phenomenon may be because a few questions posted by users in their initial search for knowledge attract much attention and gain many fans. However, as the number of fans increases, the knowledge shared by the user will be read by many fans, which will quickly lead to questioning or contradiction if the shared knowledge has errors. In other words, users will be in the environment of “Great winds blow upon high hills”, which means that “the more you say, the more you are wrong, and the more you lose”. 

In addition, the data analysis showed that reputation had a negative impact on knowledge contribution, which contradicts previous studies [62]. This phenomenon among Zhihu community users may be because their expected reward for contributing knowledge is not the “Excellent Answer” title, but rather social support, such as care, backing, and encouragement. Future studies could explore data from other community platforms to further examine the role of reputation as a motivator.

### 4.2. Theoretical and Managerial Implications

This study made three key contributions. First, while existing research primarily focuses on individual behavioral states, such as knowledge seeking or knowledge contribution, it rarely explores the connection between the two. This study examined the impact of knowledge seeking on knowledge contribution in Q&A communities, providing deeper insights into user behavior in these platforms. Second, the findings revealed that knowledge seeking influences knowledge contribution through user motivation, indicating that motivation mediates the relationship between these two behaviors. This helped clarify the process and internal mechanism of user behavior conversion in Q&A communities. Third, social support was found to be the most significant factor influencing knowledge contribution, while reputation did not have a positive effect. These results suggest that fostering a supportive environment is more important for encouraging knowledge contribution than focusing on individual reputation within Q&A communities.

These outcomes have several managerial implications for Q&A communities. First, social Q&A communities should create a supportive environment for users. For example, the platform can optimize its interface design by diversifying the presentation of user likes. Additionally, the community could offer material rewards, such as vouchers and memorabilia, to users with a high number of likes, encouraging expert users to provide valuable solutions for others. Ultimately, these measures would enhance the sense of social support that users feel from the community. Second, platforms should guide users in the right direction to increase their altruism and self-efficacy. On the one hand, they can foster a positive, friendly atmosphere by expanding promotional efforts that highlight the value of altruism. On the other hand, the platform can improve its search functionality and recommend relevant results based on users’ needs. These actions will enhance user engagement, increase the flow of knowledge, and promote the sustainable development of social Q&A platforms.

### 4.3. Limitations and Future Directions

This study had the following limitations. First, our data were only obtained from one Q&A community, Zhihu. To generalize our findings to other Q&A communities, we will investigate multiple Q&A communities, such as Baidu Post Bar and GitHub, in future studies. Second, user motivation includes a variety of factors, but we only examined five. In future studies, we will explore the possible impact of other motivational factors, such as enjoyment and reciprocity. Third, we only used quantitative methods to conduct the study and did not use qualitative methods to analyze the content of the posts. In future research, to further enhance the persuasiveness of our findings, we will consider conducting more in-depth research using a mixed research method combining qualitative and quantitative methods. Fourth, we proposed some hypotheses about the impact of knowledge seeking on knowledge contribution and collected cross-sectional data to validate these research hypotheses. The use of time series data can better validate our proposed hypotheses. Therefore, in future research, we will consider collecting time series data to conduct more in-depth research and obtain more accurate conclusions.

## 5. Conclusions

Based on motivational theory, this study examined effect of users’ knowledge seeking on users’ knowledge contribution in Q&A Communities. We collected objective data from China’s largest Q&A platform, Zhihu, and conducted the mediation effect test. The results showed that knowledge seeking affects knowledge contribution through user motivation. Among them, knowledge seeking can significantly positively affect users’ intrinsic motivation (altruism and self-efficacy) and extrinsic motivation (social support, group identity, and reputation). Altruism, self-efficacy, and social support significantly and positively affect knowledge contribution, and group identity and reputation significantly and negatively affect knowledge contribution. In addition, we found that social support plays a major role in five parallel mediators. This study not only contributed to research in the information behavior field and enriched the theoretical understanding of this topic but also provided helpful suggestions for developing Q&A platforms and valuable references for administrators.

## Figures and Tables

**Table 1:** Descriptive statistical results.

Variable	Min.	Max.	Mean	Std.
KS	2	6028	41.46	191.13
AL	0	20,611	330.15	996.05
SE	0	10,538	97.86	408.63
GI	1	12,481,671	94,466.52	298,380.87
RE	0	11	0.96	1.25
SS	0	16,116,633	177,278.57	543,086.74
KC	2	35,409	560.15	1248.80

**Table 2:** The direct effect values of each variable and analysis results.

Regression Equation		Overall Fitting Index			Significance of Regression Coefficients	
Outcome	Predictive Variables	*R*	*R* ^2^	*F*	*β*	*t*
AL	KS	0.250	0.062	308.295	0.250	17.558 ***
SE	KS	0.180	0.032	155.416	0.180	12.467 ***
SS	KS	0.191	0.037	176.389	0.191	13.281 ***
GI	KS	0.228	0.052	254.005	0.228	15.938 ***
RE	KS	0.050	0.003	11.451	0.050	3.384 ***
KC		0.755	0.570	1024.377		
	KS				0.161	16.016 ***
	AL				0.181	14.529 ***
	SE				0.126	10.120 ***
	SS				0.815	40.158 ***
	GI				−0.362	−17.308 ***
	RE				−0.020	−2.039 *

Notes: * *p* < 0.05, *** *p* < 0.001; *β* is standardized.

**Table 3:** Mediation effect.

	Relationships	Mediation Effect	SE	95% CI	Formula	Proportion of Effect
Total indirect effect		0.140	0.013	0.142~0.194		
Indirect effect	KS → AL → KC	0.045	0.005	0.044~0.065	a/b	14.95%
	KS → SE → KC	0.023	0.004	0.020~0.035	a/b	7.64%
	KS → SS → KC	0.156	0.014	0.159~0.215	a/b	51.69%
	KS → GI → KC	−0.083	0.008	−0.115~−0.084	|a/b’|	51.55%
	KS → RE → KC	−0.001	0.001	−0.003~0.000	|a/b’|	0.62%

Notes: a = Mediation effect, b = 0.301 = total effect KS on KC, b’ = 0.161 = direct effect KS on KC; mediation effect value is standardized.

**Table 4:** Reverse mediation effect.

	Relationships	Mediation Effect	SE	95% CI
Total indirect effect		−0.025	0.015	−0.056~0.006
Indirect effect	KC → AL → KS	0.065	0.011	0.044~0.087
	KC → SE → KS	−0.014	0.009	−0.032~0.003
	KC → SS → KS	−0.252	0.024	−0.299~−0.204
	KC → GI → KS	0.175	0.018	0.140~0.211
	KC → RE → KS	0.001	0.001	−0.001~0.002

Notes: mediation effect value is standardized.

## Data Availability

The data presented in this study are available on request from the corresponding author.

## References

[1] Wang N., Wang L., Ma Z., Wang S. (2022). From knowledge seeking to knowledge contribution: A social capital perspective on knowledge sharing behaviors in online Q&A communities. Technol. Forecast. Soc. Chang..

[2] Luo C., Lan Y., Luo X.R., Li H. (2021). The effect of commitment on knowledge sharing: An empirical study of virtual communities. Technol. Forecast. Soc. Chang..

[3] Zhou T., Mi Q. (2023). Examining user switching between social Q&A platforms: A push–pull-mooring perspective. Univers. Access Inf. Soc..

[4] Zhao X., Zhao L., Wu T., Zhang Z. (2022). Research on multiple mediating effects of the transformation from serchers to contributors in online knowledge community. J. Modern Inf..

[5] Elisabeth J., Kraut R.E. (2006). Predicting continued participation in newsgroups. J. Comput. Mediat. Commun..

[6] Luo N., Wang Y., Zhang M., Niu T., Tu J. (2020). Integrating community and e-commerce to build a trusted online second-hand platform: Based on the perspective of social capital. Technol. Forecast. Soc. Chang..

[7] Zhou T., Ye Z. (2022). A research on the knowledge contribution behaviour of open source community users based on the social cognitive theory. Eval. Manag..

[8] Deng S., Xia S., Xu J., Fu S. (2022). Research on factors affecting knowledge contribution behavior of physicians in online heath community from the configuration perspective. Inf. Stud. Theor. Appl..

[9] Feng X., Wang X., Xue Y., Yu H. (2023). Analysis of the characteristics and evolution of knowledge label networks in the Q&A community: Taking the Zhihu platform as an example. Electron. Lib..

[10] Zhao Y., Xuan X., Li L., Zhao Y. (2018). The impact factors of users’ paying behavior for knowledge on social Q&A platform based on social capital theory. Doc. Inf. Knowl..

[11] Zhao Y., Liu Z., Zhu Q. (2020). From free to fee: Exploring askers. J. Chin. Soc. Sci. Technol. Inf..

[12] Li M., Liang J. (2024). Two-way deviation: The impact of the deviation of horizontal and vertical answers on knowledge adoption in virtual Q&A communities. Lib. Hi Tech.

[13] Zhang Y., Zhu Q. (2018). Answerer selection behavior of questioner in paid knowledge Q&A community. Inf. Stud. Theor. Appl..

[14] Li L., Li AR Z., Song X., Li X.R., Huang K., Ye E.M. (2023). Characterizing response quantity on academic social Q&A sites: A multidiscipline comparison of linguistic characteristics of questions. Lib. Hi Tech.

[15] Hubley C., Edwards J., Miele D.B., Scholer A.A. (2024). Metamotivational beliefs about intrinsic and extrinsic motivation. J. Personal. Soc. Psychol..

[16] Meena R., Sarabhai S. (2023). Extrinsic and intrinsic motivators for usage continuance of hedonic mobile apps. J. Retail. Consum. Serv..

[17] Mercader-Rubio I., Ángel N.G., Silva S., Furtado G., Brito-Costa S. (2023). Intrinsic motivation: Knowledge, achievement, and experimentation in sports science students—Relations with emotional intelligence. Behav. Sci..

[18] Messerer LA S., Karst K., Janke S. (2023). Choose wisely: Intrinsic motivation for enrollment is associated with ongoing intrinsic learning motivation, study success and dropout. Stud. Higher Educ..

[19] Barbieri V., Wiedermann C.J., Lombardo S., Plagg B., Piccoliori G., Gärtner T., Engl A. (2023). Age-related associations of altruism with attitudes towards COVID-19 and vaccination: A representative survey in the North of Italy. Behav. Sci..

[20] Chen X., Zhang X., Zeng S., Hu M. (2017). The factors of knowledge sharing intention in the health Q&A communities. J. Modern Inf..

[21] Yin M., Li Q. (2017). Understanding users’ participation intention of microblog topics based on herd behavior and motivation theory. Inf. Sci..

[22] Geng R., Shen J. (2019). Research on SNS users’ knowledge sharing motivation from different cultural perspectives. J. Lib. Sci. Chin..

[23] Cao F., Zhang J., Wang J., Liu K., Yang H. (2019). Study on the influencing factors of users’ adaptive academic information seeking behavior in academic search engines. J. Natl. Lib. Chin..

[24] Wang L., Kim K. (2024). Fostering knowledge exchange in digital communities: Psychological determinants of sharing in Q&A platforms. J. Knowl. Econ..

[25] Burmeister A., Song Y., Wang M., Hirschi A. (2024). Understanding knowledge sharing from an identity-Based motivational perspective. J. Manag..

[26] Mitchell R., Schuster L., Jin H.S. (2020). Gamification and the impact of extrinsic motivation on needs satisfaction: Making work fun?. J. Bus. Res..

[27] Keshavarz H., Vafaeian A., Shabani A. (2023). Toward the dialectical evaluation of online information: The roles of personality, self-efficacy and attitude. Lib. Hi Tech.

[28] Zhao Y., Liu Z., Song S. (2018). Exploring the influential factors of askers’ intention to pay in knowledge Q&A platforms. Data Anal. Knowl. Discov..

[29] Shanmugam M., Sun S., Amidi A., Khani F., Khani F. (2016). The applications of social commerce constructs. Int. J. Inf. Manag..

[30] Guo Y., Lu Z., Wang C. (2021). The impact mechanism of peer characteristics on user’s social sharing intention in the social commerce context. J. Intell..

[31] Zhou T., Yang W. (2020). Study of online health community users knowledge sharing behaviors based on social influence theory. J. Inf. Manag..

[32] Wang M. (2013). Research on Critical factors of the behavior of obtaining online health information in America. J. Inf. Resour. Manag..

[33] Liu Z., Li W., Huang Y. (2022). Network community learning effect: Theoretical mechanism and empirical test. Manag. Rev..

[34] Lee J.H., Jung S.H., Park J.H. (2017). The role of entropy of review text sentiments on online WOM and movie box office sales. Electron. Commer. Res. Appl..

[35] Cui Z., Tu Y. (2020). Research on continuous contribution behavior of paid knowledge based on perceived value and motivation theory—Taking Zhihu Live as an example. Knowl. Manag. Forum.

[36] Liu H., Li Y. (2020). Research on influence factors of knowledge sharing intention of academic social network users. J. Modern Inf..

[37] Zhou T., Zeng H., Deng S. (2019). The Research on the effect of information privacy concern in the context of social commerce. J. Modern Inf..

[38] Zhang T., Wang WY C., Techatassanasoontorn A.A. (2019). User’s feedback contribution to enhance professional online community: A motivational process. VINE J. Inf. Knowl. Manag. Syst..

[39] Nguyen T.M., Ngo L.V., Gregory G. (2022). Motivation in organisational online knowledge sharing. J. Knowl. Manag..

[40] Qin H., Wang H., Johnson A. (2020). Understanding the information needs and information-seeking behaviours of new-generation engineering designers for effective knowledge management. Aslib J. Inf. Manag..

[41] Veeravalli S., Venkatraman V., Hariharan M. (2020). Why do people seek knowledge? Tracing factors that affect knowledge seeking intention. VINE J. Inf. Knowl. Manag. Syst..

[42] Fu S., Chen X., Deng S. (2017). Information behavioral transferring in a social Q&A community: A conceptual model for understanding mechanism from lnformation adoption to sustained information seeking. Doc. Inf. Knowl..

[43] Jin J., Zhang T., Yan X. (2023). Why do users continually seek knowledge in online Q&A communities? An empirical investigation. Inf. Discov. Deliv..

[44] Jiang S., Nguyen D.K., Dai P.F., Meng Q. (2024). Monetary income as opportunity cost: Exploring the negative effect on free knowledge contribution of knowledge suppliers. J. Knowl. Manag..

[45] Wang N., Wang L., Li Y., Chen J. (2021). The effect of peer influence on users’ contribution behavior in online innovation community—Analysis based on network objective data. Stud. Sci. Sci..

[46] Zhou Y., Zhu L., Wu C., Wang H., Wang Q., Yuan Q. (2023). Effects of social media affordances on knowledge contribution in online Q&A communities: A self-determination perspective. Ind. Manag. Data Sys.

[47] Luo L., Wang Y., Duan S., Shang S., Ma B., Zhou X. (2024). Continuous knowledge contribution in social Q&A communities: The moderation effects of self-presentation and motivational affordances. Inf. Technol. People.

[48] Zhang M., Xue Y.X., Yang J., Zhang Y. (2023). Why should I contribute my voice? Analysis of members’ knowledge contribution behavior from a perspective of social distance. Lib. Hi Tech.

[49] Park J.H., Gu B., Leung AC M., Konana P. (2014). An investigation of information sharing and seeking behaviors in online investment communities. Comput. Hum. Behav..

[50] Hung S.Y., Durcikova A., Lai H.M., Lin W.M. (2011). The influence of intrinsic and extrinsic motivation on individuals’ knowledge sharing behavior. Int. J. Hum. Comput. Stud..

[51] Cheung CM K., Lee MK O., Lee ZW Y. (2013). Understanding the continuance intention of knowledge sharing in online communities of practice through the post-knowledge-sharing evaluation processes. J. Am. Soc. Inf. Sci. Technol..

[52] Le T.D. (2018). Influence of WOM and content type on online engagement in consumption communities. Online Inf. Rev..

[53] Farivar S., Wang F. (2022). Effective influencer marketing: A social identity perspective. J. Retail. Consum. Serv..

[54] Koski J.E., Collins J.A., Olson I.R. (2017). The neural representation of social status in the extended face-processing network. Eur. J. Neurosci..

[55] Xu Y., Liu H., Liu H. (2024). Parallel mediating role of technology intrusion and fear of negative evaluation in the relationship between social anxiety and smartphone addiction of college students. Chin. J. Health Psychol..

[56] Li M.N., Ren Y.L., Liu L.J., Cheng M.H., Di Q., Chang H.J., Li Q., Wang L.N., Ma A. (2024). The effect of emotion regulation on empathic ability in Chinese nursing students: The parallel mediating role of emotional intelligence and self-consistency congruence. Nurse Educ. Pract..

[57] Hayes A.F. (2018). Introduction to Mediation, Moderation, and Conditional Process Analysis: A Regression-Based Approach.

[58] Xie Y. (2010). Regression Analysis.

[59] Wasko M.M., Faraj S. (2005). Why should I share? examining social capital and knowledge contribution in electronic networks of practice. MIS Q..

[60] Shankar R., Wang L., Gunasti K., Li H. (2024). Nonverbal peer feedback and user contribution in online forums: Experimental evidence of the role of attribution and emotions. J. Assoc. Inf. Syst..

[61] Zhou T. (2021). Examining online health community users’ sharing behaviour: A social influence perspective. Inf. Dev..

[62] Zhang X., Liu S., Deng Z., Chen X. (2017). Knowledge sharing motivations in online health communities: A comparative study of health professionals and normal users. Comput. Hum. Behav..

